# *ATfiltR*: A solution for managing and filtering detections from passive acoustic telemetry data

**DOI:** 10.1016/j.mex.2023.102222

**Published:** 2023-05-14

**Authors:** Félicie Dhellemmes, Eneko Aspillaga, Christopher T. Monk

**Affiliations:** a Department of Fish Biology, Fisheries and Aquaculture, Leibniz Institute of Freshwater Ecology and Inland Fisheries, Berlin, Germany; b Instituto Mediterráneo de Estudios Avanzados, IMEDEA (CSIC-UIB), Spain; c GEOMAR Helmholtz-Centre for Ocean Research, Kiel, Germany

**Keywords:** Acoustic telemetry detection filter with ATfiltR, Acoustic telemetry, Ghost detections, Spurious detections, Tag collisions, Detection filter, Data management

## Abstract

Acoustic telemetry is a popular and cost-efficient method for tracking the movements of animals in the aquatic ecosystem. But data acquired via acoustic telemetry often contains spurious detections that must be identified and excluded by researchers to ensure valid results. Such data management is difficult as the amount of data collected often surpasses the capabilities of simple spreadsheet applications. *ATfiltR* is an open-source package programmed in R that allows users to integrate all telemetry data collected into a single file, to conditionally attribute animal data and location data to detections and to filter spurious detections based on customizable rules. Such tool will likely be useful to new researchers in acoustic telemetry and enhance results reproducibility.

• *ATfiltR* compiles telemetry files and identifies and stores all data that was collected outside of your study period (e.g. when your receivers were on land for servicing) elsewhere.
• As spurious detections are unlikely to appear sequentially in the data, *ATfiltR* finds all detections that occurred only once (per receiver or in the whole array) within a user-designated time period and stores them elsewhere.
• *ATfiltR* identifies detections that are impossible given the animals’ swimming speeds and the receivers detection range and stores them elsewhere.

## Graphical abstract

**Figure:**
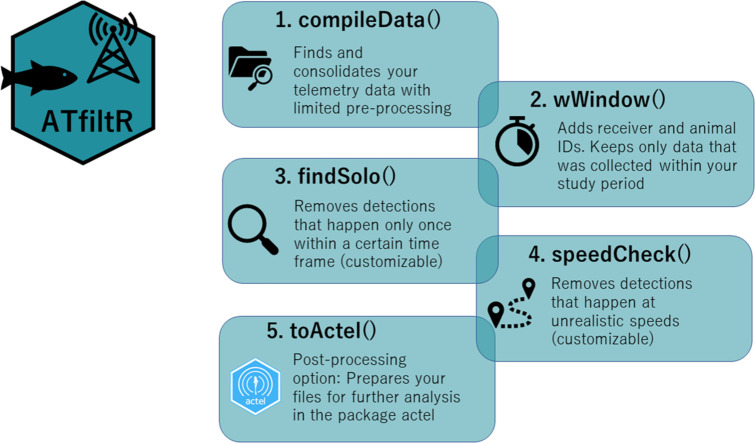

Specifications Table

**Table:** 

Subject area:	Agricultural and Biological Sciences
More specific subject area:	Animal movements
Name of your method:	Acoustic telemetry detection filter with ATfiltR
Name and reference of original method:	**Necessity for filtering:** Simpfendorfer, C. A., Huveneers, C., Steckenreuter, A., Tattersall, K., Hoenner, X., Harcourt, R., & Heupel, M. R. (2015). Ghosts in the data: False detections in VEMCO pulse position modulation acoustic telemetry monitoring equipment. Animal Biotelemetry, 3(1), 1–10. https://doi.org/10.1186/s40317-015-0094-z
	**Some descriptions of filtering protocol:** Gupte, Pratik Rajan, Christine E. Beardsworth, Orr Spiegel, Emmanuel Lourie, Sivan Toledo, Ran Nathan, and Allert I. Bijleveld. “A Guide to Pre‐processing High‐throughput Animal Tracking Data.” Journal of Animal Ecology 91, no. 2 (February 2022): 287–307. https://doi.org/10.1111/1365-2656.13610. Kessel, S. T., Chapman, D. D., Franks, B. R., Gedamke, T., Gruber, S. H., Newman, J. M., White, E. R., & Perkins, R. G. (2014). Predictable temperature-regulated residency, movement and migration in a large, highly mobile marine predator (*Negaprion brevirostris*). Marine Ecology Progress Series, 514, 175–190. https://doi.org/10.3354/meps10966 **An alternative and complementary filtering tool:** Flávio, H., & Baktoft, H. (2021). actel: Standardised analysis of acoustic telemetry data from animals moving through receiver arrays. Methods in Ecology and Evolution, 12(1), 196–203. https://doi.org/10.1111/2041-210X.13503
Resource availability:	https://github.com/FelicieDh/ATfiltR

## Method details

### Background

Acoustic telemetry has become a key method in the field of aquatic movement ecology thanks to its cost efficiency and the spatial and temporal resolutions at which it allows organisms to be tracked, even in difficult to access habitats [10]. Acoustic telemetry uses underwater acoustic receivers, enabling researchers to record the local presence and sometimes exact location of animals fitted with transmitters that emit encoded acoustic signals (i.e. acoustic tags) [8]. While the hardware and sampling methods differ across studies, acoustic telemetry can be broadly classified into two categories: active acoustic telemetry, where animals are tracked in real-time by a mobile receiver (e.g. from a boat); and passive acoustic telemetry, where several remote receivers are deployed underwater at fixed positions and continuously listen and record detections and related data which can regularly be downloaded. Passive acoustic telemetry is broadly favored as it allows tracking animals around the clock, independent of the presence of researchers on site, and has an excellent ratio of data acquired to labor intensity [8]. But passive acoustic receivers have been known to record false positives (i.e. false or spurious detections; [13]), which can significantly impact the interpretation of the results [2]. As a result, data handling and filtering is a critical step for passive acoustic telemetry studies [6,7]. Because data is acquired continuously, traditional spreadsheet applications are usually insufficient to handle datasets, and this has to be done using algorithms in programming languages instead, which is an especially time-consuming task [7].

In this context and to improve the reproducibility of passive acoustic telemetry studies, the package *actel* was released for R in 2021 [4,12]. *actel* allows users to process and filter passive acoustic telemetry data in a systematic and reproducible way (among other things) and, naturally, we turned to this solution for our own data (Dhellemmes et al. under review, Fisheries Research). Unfortunately, with over 29 million data points collected from more than 300 animals at 145 geographical locations, we found *actel* not to be a perfectly appropriate solution in our case. User input is central to *actel*, which identifies potentially problematic detections in the data, displays them and lets the user decide whether detections should be erased. While this allows for a very fine-tuning of the filtering process, it can become extremely time-consuming when datasets are large, and user decisions might be inconsistent over time, leading to potential biases. Here, we present *ATfiltR*, an alternative open-source R package that processes passive telemetry datasets and filters spurious detections according to a fully customizable set of rules. The package consists of a suite of five functions, one of which allows the user to prepare their data following processing in *ATfiltR* for use in *actel*, allowing researchers to potentially use *ATfiltR* for coarse batch processing and *actel* for finer filtering and data exploration.

### Description of the tool

*ATfiltR* consists of five functions, each described below, that can be used consecutively to handle and filter passive acoustic telemetry data. The five functions in *ATfiltR* are designed to be used in sequential order, and will respectively, (1) load and organize raw detection files, (2) load and organize animal meta data and receiver deployment files, while attributing this information to the detection data and identifying detections outside of receiver deployments, (3) identify and filter unlikely solitary detections within a specified duration, (4) identify and filter detections that occur at impossible swimming speeds and 5) prepare the filtered dataset for further analysis with the *actel* package.

One important detail is that *ATfiltR* is mostly project-based. This means that it is meant to operate within an R Studio project [1] which is an automatic way to set the root directory in which the work should be performed. This allows *ATfiltR* to be directly used across machines and collaboratively (e.g. through cloud-based directories) without having to amend the working directory.

Two of the functions (*findSolo()* and *speedCheck()*, described below) can be used outside of a project, if users are only interested in using one or both independently of the rest of the available functions.

*ATfiltR* was partly created using the *data.table* syntax [3], an alternative to the native *R* syntax allowing for higher processing speed, specifically for large datasets.

### compileData()

**Fig. 1:**
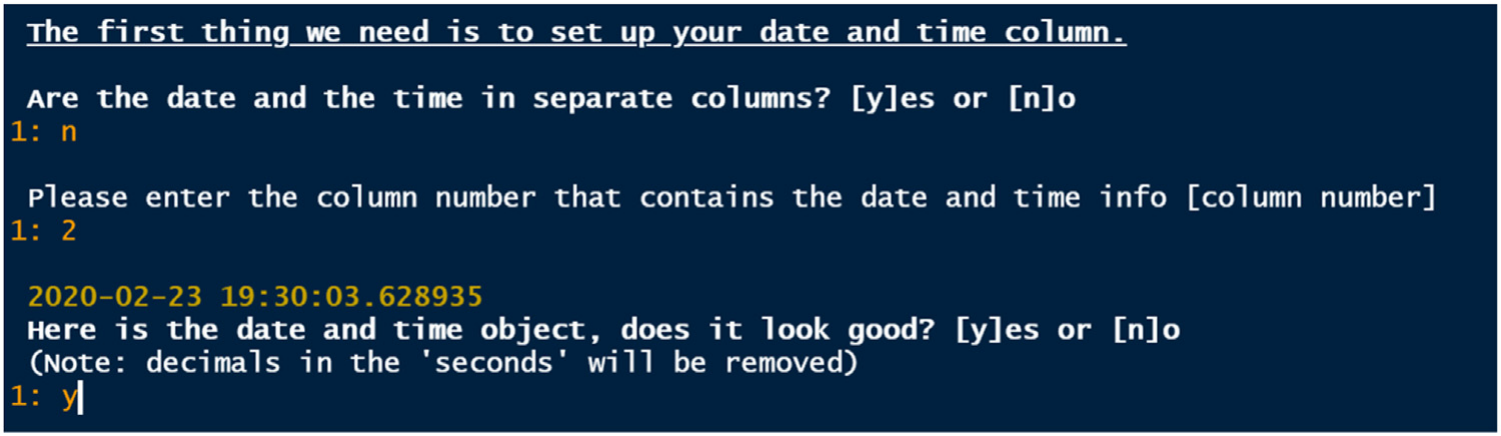
Example screenshot of the user-input process used in *compileData()* to identify relevant columns in the data. Here, data and time.

This function identifies all data files that have the extension indicated by *file.ext* within the folder indicated by *detection.folder* (Table 1). If files are found, one is loaded in R, and a dialogue with the user starts to identify key features in the data (Fig. 1): (1) the row in which the column names are stored (with the option for the user to input their own column names), (2) whether some of the first rows should be omitted from the data, (3) whether some of the columns should be omitted from the data, (4) whether the date and the time are in separate columns (and then in which column are dates and times stored), (5) which column contains the IDs of the transmitters, (6) which column contains the ID of the receiver. This is done because different acoustic telemetry systems may have slightly different formats, and we wanted *ATfiltR* to be useable across platforms with minimal reformatting required from the user. The format of the time and date is especially critical on such spatio-temporal data, and the integration of *lubridate*’s parse_date_time function allows for a variety of different formats to be handled [5]. Once the dialogue is over, *ATfiltR* loads all the other data files (in batches if *split=T*), formats them, and compiles them into one single RData file, which is saved in the *detection.folder* if *save=T*. The user also has the option of letting ATfiltR identify duplicated data points (detections of the same transmitter ID, on the same receiver at the same date and time), removing them and saving them in a separate file.

**Table 1:** Arguments used in the different *ATfiltR* functions.

Argument	Description	compileData()	wWindow()	findSolo()	speedCheck()	toActel()
detection.folder	Character string. The name of the folder containing the detection data files.	**x**		**x**	**x**	**x**
data.folder	Character string. The name of the folder containing the other data files (deployment, spatial and animal data). Can be the same as detection.folder.		**x**		**x**	**x**
file.ext	Character string. The extension of the detection data files stored in the detection.folder.	**x**				
sep.type	Character string. The character that delimits columns in the detection data files (for compileData) and in the other data files (for wWindow).	**x**	**x**			
save	TRUE or FALSE. Should the data be saved in the detections.folder post-processing? If FALSE, the data is only in the R environment.	**x**	**x**	**x**	**x**	
remove.duplicates	TRUE or FALSE. Should the duplicates found in the data be removed?	**x**				
save.duplicates	TRUE or FALSE. Should the duplicates be saved in the detections.folder post processing?	**x**				
split	TRUE or FALSE. Should the data be compiled in small batches? This is recommended for large data files, as it prevents the software from running out of memory.	**x**				
save.out.of.deployment	TRUE or FALSE. Should the data obtained outside the deployment period be saved in the detections.folder post processing?		**x**			
save.unknown.tags	TRUE or FALSE. Should the data from unknown tags be saved in the detections.folder post processing?		**x**			
discard.first	Numeric. How many of the first hours after deploying a tag should be discarded? (e.g. 24 = the first 24h will be discarded). If save.unknown.tags is TRUE, the discarded data will be saved in the unknown tags file.		**x**			
save.solo	TRUE or FALSE. Should the solitary detections be saved in the detections.folder post processing?			**x**		
per.receiver	TRUE or FALSE. If TRUE (default), the solitary detections are considered solitary when they occur alone on a given receiver (Similarly to Kessel et al. 2014). If FALSE, they are considered solitary when they occur alone across the whole array.			**x**		
delay	Numeric (hours). Defines solitary detections: detections that are recorded *delay* hours after the previous one and *delay* hours before the subsequent one are considered solitary.			**x**		
receiver.range	Numeric. Range of the receivers in meters (if the range is the same for all receivers and the whole study duration). Keep as NA if the range data is stored in a separate file (i.e. if different receivers have different ranges).				**x**	
base	Numeric. The base of the equation used for speed calculation (in m/s). For instance, if the speed is calculated as speed=2*body_length^0.015, the base is 2. If the speed is the same for all fish, for instance 10m/s, the base is 10.				**x**	
factor.col	Character. The column name for the data to use in the speed calculation. For instance, if speed=2*body_length^0.015, “factor.col” is the name of the column that contains the necessary body length data, in quotation marks “body_length”. If the speed does not depend on data in your dataset, keep as NA.				**x**	
exponent	Numeric. The exponent for the factor.col you indicated in your speed calculation. For instance, for speed=2*body_length^0.015, exponent is equal to 0.015				**x**	
max.distance	Numeric. Maximum distance (in meters) that an animal may move beyond which the detections should be removed. Default: NA				**x**	
save.speedy	TRUE or FALSE. Should the detections that were removed via the speed filter be saved in the detections.folder post processing?				**x**	
target.folder	Character string. The name of the folder in which the data should be stored once prepared for *actel*. Can be the same as the detection.folder or the data.folder.					**x**
project	TRUE or FALSE. Should ATfiltR work within an R studio project?			**x**	**x**	
data.file	Character. The name of the R object containing the data to be used (only if project=F)			**x**	**x**	
ID.col	Character. The name of column containing the animal ID (only if project=F)			**x**	**x**	
DateTime.col	Character. The name of column containing the timestamp (only if project=F)			**x**	**x**	
Station.col	Character. The name of column containing the station ID (only if project=F)			**x**	**x**

### wWindow()

*wWindow* uses the previously compiled data frame (the most recently created data frame compiled via *compileData* that is found in the *detection.folder* is automatically loaded) and attributes animal data and receiver location data to the data frame. To do that, the user needs to provide three files that are saved in the *data.folder* (Table 1). Everyone handles and enters data slightly differently, so we designed *ATfiltR* to work with user input to identify which data sets to use, what columns contain the relevant information etc. This way, users can use *ATfiltR* with minimal prior reformatting of the data.

The three necessary files contain the spatial data, the deployment data, and the animal data. We will now describe the conditions these files must meet to be useable by *ATfiltR*.

Spatial data must contain a longitude column, a latitude column (in any format), a station name column which represents the unique name of each location where receivers have been deployed, and (optionally) a range category column which indicates the names of the category used when attributing each receiver's ranges (if receivers have different ranges; only relevant for future *speedCheck()*). Each row corresponds to one location (i.e. station) at which receivers have been deployed.

**Fig. 2:**
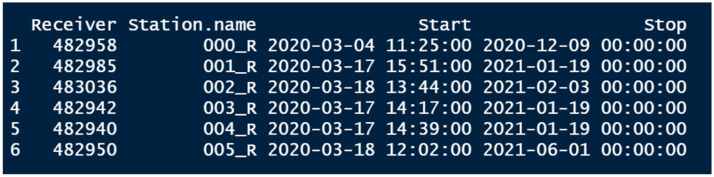
Example of the deployment data.

Deployment data must contain a column with the receiver ID (in the same format as in the detection data), the name of the location at which it is deployed (station name, corresponding to the names in the spatial data file), the date and time at which a receiver is deployed, and the date and time at which it is retrieved (Fig. 2). Each row corresponds to one deployment event (from deployment to retrieval) for a receiver. Receivers that are redeployed multiple times require multiple rows.

Animal data must contain a transmitter ID column (in the same format as in your detection data), a unique animal ID column (to allow transmitters to be deployed consecutively in multiple animals), and a date and time column (date and time of tag implantation). Each row corresponds to the tag implantation of one animal. If there are more rows per individual (e.g. recapture events), users may indicate a tag.status column to keep track of the events. If the animal data includes a column with the name of the location where it was captured, the column can also be identified.

**Fig. 3:**
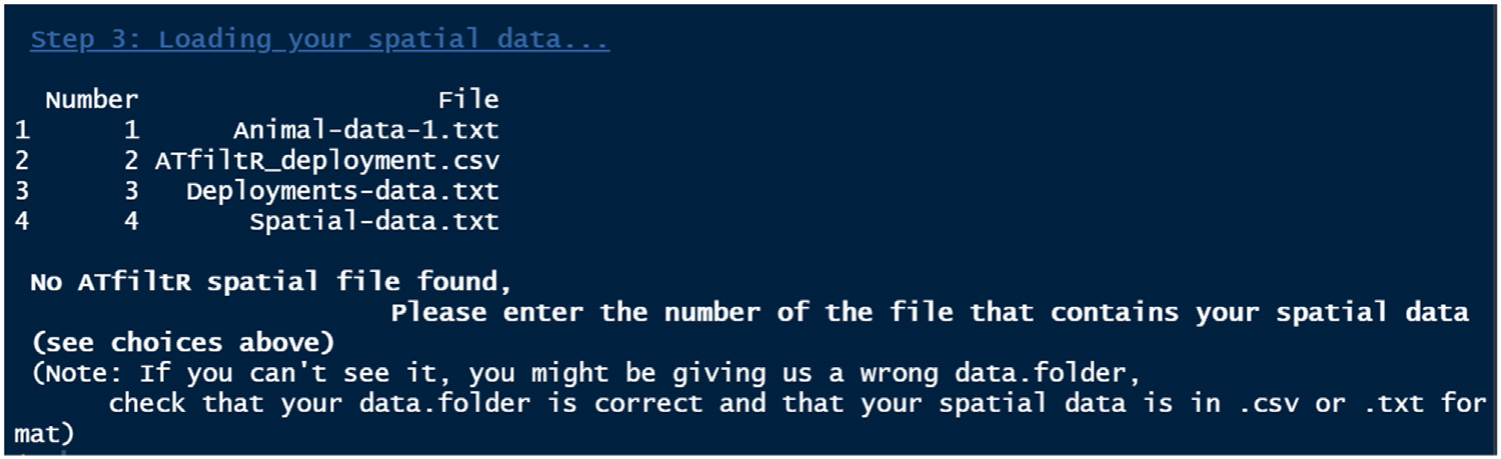
Example of a user-input event. The names of the files do not matter, as the program will ask the user to identify the file that they wish to use each time.

All data files may contain more columns than the necessary ones. The names of the files and the names of the columns do not matter, as users may choose the files they wish to use (Fig. 3) and will indicate which columns are relevant. Once this is done, the *ATfiltR* saves the three data files in a standard format in the *data.folder*. This means that the function can be used again without needing any user input: *AtfiltR* will automatically load the standard files and proceed with the data processing.

*wWindow* then attributes the receiver station names, longitude and latitude data to all rows, and identifies data that is collected outside of a deployment “window” (the time span between the deployment and the retrieval of a receiver).

Similarly, the animal IDs are attributed, and any data collected from an undeployed transmitter (or from other transmitters in the area which are not included in the animal data) is identified. Other columns (e.g. body size) in the animal file may be also added to the detection data.

The data is then saved in a new, timestamped RData file, which is saved in the *detection.folder* if *save=T*.

### findSolo()

When animals occur within the range of a receiver, they are usually there long enough to log multiple detections on that receiver. Consequently, spurious detections are commonly identified as detections that are recorded only once within a certain time frame (i.e. solitary detections). The time frame used and whether the detection should be detected only once on a given receiver or on the whole array differs from study to study. For instance, Meyer et al. [11] identified spurious detections as detections recorded only once on the whole array during a 24h time frame. Kessel et al. [9] considered detections that occurred only once on a given receiver within a 1h period as spurious.

**Fig. 4:**
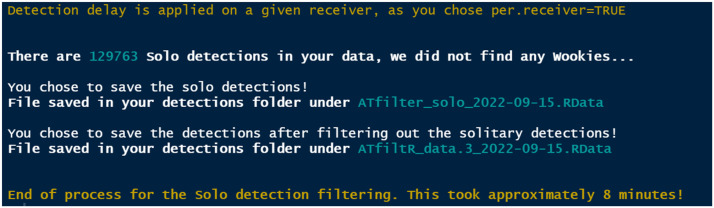
Example output of the *findSolo()* function with *per.receiver=T, save.solo=T* and *save=T*.

*findSolo* is a fully customizable rule-based tool allowing researchers to identify spurious detections (Fig. 4). It uses the data previously generated via *compileData* and *wWindow* (and automatically loads the most recent file). The user can indicate whether solitary detections should be considered on a per receiver basis or over the whole array with *per.receiver* (TRUE or FALSE). The time frame can also be indicated by using the *delay* argument (Table 1).

Once the solitary detections are found, the user has the option to save them separately (*save.solo*=TRUE). The data is then saved in a new, timestamped RData file, which is saved in the *detection.folder* if *save=T*.

This function can be used outside of an R studio project by indicating project=F and the appropriate *data.file* (object that contains the data to be used), *ID.col* (name of the column containing the animal ID), *DateTime.col* (name of the column containing the timestamp) and *Station.col* (name of the column containing the location ID).

### speedCheck()

Spurious detections may also be identified if they are logged on receivers that are at a distance that animals could not feasibly travel from their previous location within the time frame at which they were logged. *speedCheck* uses the distance between receivers, the theoretical swimming speed of the animals (customizable) and the receiver range (customizable) to estimate the feasibility of detections.

The function requires a distance matrix among the stations with the column names and the row names in the matrix corresponding to each station name in the spatial data file. If no landmasses are present in the study area, users may use the Haversine formula to calculate the matrix themselves. Otherwise, we recommend using *actel::distancesMatrix()* with *actel=F* from the spatial data file [4].

If receivers have different ranges, these may be attributed using a range file containing a column with the range category, time step (if range varies through time) and the range in meters.

**Fig. 5:**
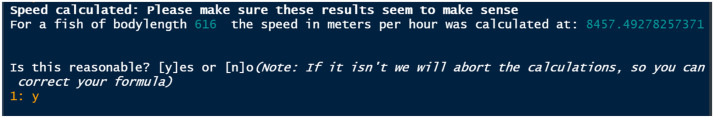
Example dialogue with the program ensuring that speed calculations are reasonable. Here a fish of 616mm total length is calculated to have a speed of 8457m.h^−1^ via the critical speed formula for fish speed in [14]: *base* = 0.019, *factor* = ``TL'', *exponent* = 0.75.

The speed (m.h^−1^) of each animal can be calculated via an equation, allowing speed to scale with body size for example (Fig. 5). Speed can also be the same for all animals, in the case the user can indicate *factor* = NA, *exponent* = NA (default setting) and the speed they wish to use in m.h^−1^ in the *base* argument.

**Fig. 6:**
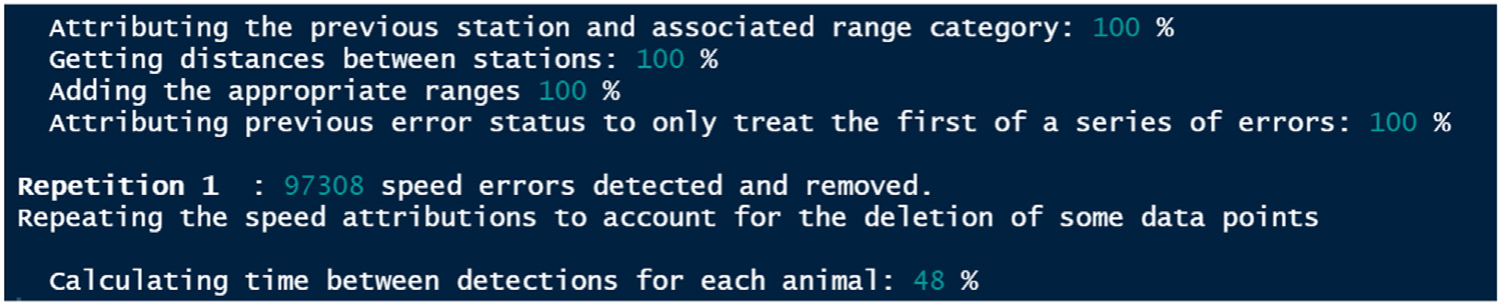
Example of the iterative process used to identify speed errors.

*speedCheck* operates by identifying detections that occur at an unreasonable speed, removing them, and reiterating this process until no detections that are logged above threshold speeds can be found (Fig. 6). The process is then repeated as many times as is necessary until all unreasonably fast detections are removed (and saved elsewhere if *save.speedy=T*).

Similary to findSolo(), this function can be used outside of an R studio project by indicating project=F and the appropriate data.file (object that contains the data to be used), ID.col (name of the column containing the animal ID), DateTime.col (name of the column containing the timestamp) and Station.col (name of the column containing the location ID).

### toActel()

*actel* requires some formatting to be used. *ATfiltR* has already identified the relevant columns in the detection file, in the animal, deployment and spatial data and so it can make the formatting for the user. Users may pick which detection file (already processed through *ATfiltR*) to use, and the function automatically creates an *actel.detection.RData* file, a *biometrics.csv*, a *deployments.csv* and a *spatial.csv* in the users’ choice directory. These files can be directly used for basic post-processing in the *actel* package, including data exploration tools.

### Test of the method

We created a realistic test dataset consisting of four animal detections across three receivers, and ran all functions successfully. Detailed code and results can be found in the Appendix.

## Conclusions

With *ATfiltR,* we were able to filter our own passive acoustic telemetry data in a fast, stable and reproducible way across different projects with minimal reformatting. *ATfiltR* can be used as a standalone solution or as a preliminary step before using the package *actel*. Because each project is different, users may not find *ATfiltR* fully compatible with their own data (just like we found our data difficult to filter in *actel*). This package is an open-source cooperative project, hosted on GitHub, and we expect changes to the main functions as user needs arise, and users may use parts of all of the code we developed as well as suggest corrections to the functions.

The publication of *ATfiltR,* as well as that of *actel* previously, will speed up the publication process of passive acoustic telemetry projects as researchers may handle their data without having to develop a proprietary algorithm. It may also improve replicability as the code used to filter the data can be easily described and published.

## Ethics statements

None.

## Funding

This work was supported by the European Maritime and Fisheries Fund and the State of Mecklenburg-Vorpommern, Ministry of agriculture and environment (Grant/Award numbers MV-I.18-LM-004 and B730117000069;BODDENHECHT).

## CRediT authorship contribution statement

**Félicie Dhellemmes:** Conceptualization, Methodology, Software, Writing – original draft. **Eneko Aspillaga:** Conceptualization, Software, Writing – review & editing. **Christopher T. Monk:** Conceptualization, Software, Writing – review & editing.

## Declaration of Competing Interest

The authors declare that they have no known competing financial interests or personal relationships that could have appeared to influence the work reported in this paper.

## Data Availability

The code and test data are shared.

## Appendix Supplementary materials

Image, application 1
