# Supplementary material for: ATfiltR: A solution for managing and filtering detections from passive acoustic telemetry data
Source: MethodsX. 2023 May 14;10:102222. doi: 10.1016/j.mex.2023.102222 (PMC10209445; doi:10.1016/j.mex.2023.102222)
Supplement: Supplementary file 1 [file mmc1.pdf]

Testing ATfiltR

Dhellemmes, Aspillaga & Monk

2022

Creating the test data

This is done within a project

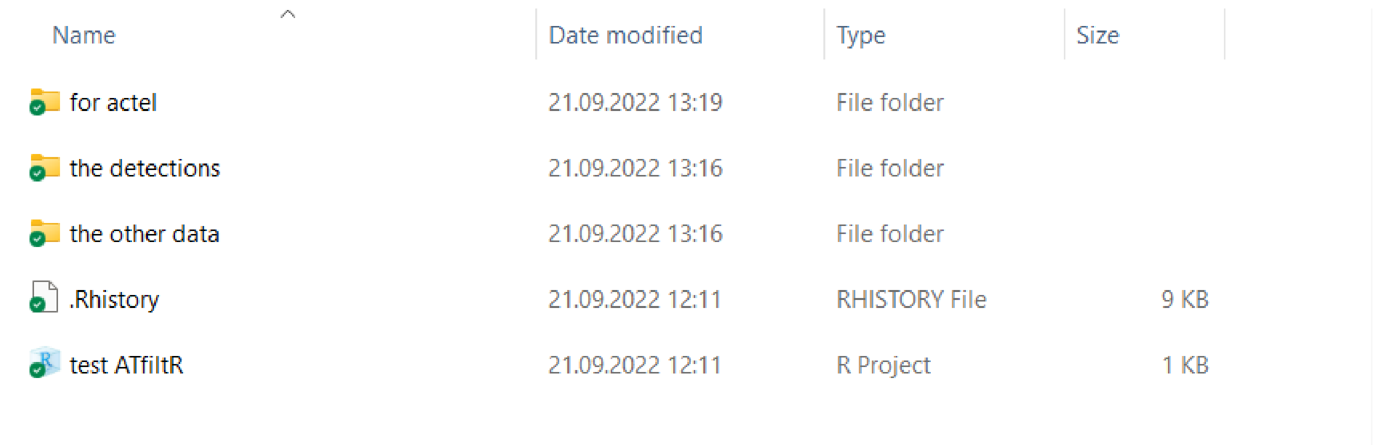

Screenshot of the project directory

Making the side data

```
set.seed(13)


fakedepl<-data.frame(receiverID = c(11111,22222,33333,22222),
                     station = c("Tatooine", "Hoth", "Endor","Tatooine"),
                     deployment.time = c("2020-10-01 12:32:11",
                                         "2020-10-01 14:25:08",
                                         "2020-10-01 12:47:05",
                                         "2020-12-04 18:24:41"),
                     retrieval.time = c("2020-12-04 11:33:02",
                                        "2020-12-04 12:02:45",
                                        "2021-03-12 06:58:47",
                                        "2021-03-12 08:03:13"))

fakeanimal<-data.frame(transmitterID = c(12365,12366,12367,12368),
                       animalID = c("Ewok1", "Ewok2", "Wookie1","Tauntaun3"),
                       Date.Time = c("2020-10-04 12:00:36",
```

```
                                    "2020-10-04 12:18:36",
                                    "2020-10-08 02:22:10",
                                    "2020-12-26 20:18:31"),
                      ship.speed= c(10000, 10000, 15000, 4000))


fakespat<-data.frame(station = c("Tatooine", "Hoth", "Endor"),
                     longitude = c(47.9255, 7.4483, -124.0046),
                     latitude = c(34.7359, 60.5477, 41.2131),
                     range.cat = c ("Tatooine+Hoth","Tatooine+Hoth","Endor"))


fakerange<-data.frame(Category = c("Tatooine+Hoth",
                                "Tatooine+Hoth",
                                "Tatooine+Hoth",
                                "Tatooine+Hoth",
                                "Tatooine+Hoth",
                                "Endor",
                                "Endor",
                                "Endor",
                                "Endor",
                                "Endor"),
                      TimeStep = c("2020-11","2020-12",
                                  "2021-01","2021-02",
                                  "2021-03","2020-11",
                                  "2020-12","2021-01",
                                  "2021-02","2021-03"),
                      Range = c(1000, 1100, 900, 1000, 1200,
                               300,500, 600, 200, 800))

fakedist<-data.frame(Tatooine = c(0, 4066769, 11542686),
                     Hoth = c(4066769, 0, 7881555),
                     Endor = c(11542686, 7881555, 0))
row.names(fakedist)<-c("Tatooine","Hoth","Endor")


write.table(fakeanimal,
            file=here::here("the other data","creatures.txt"),
              sep=",", row.names=F)

write.table(fakespat,
            file=here::here("the other data","places.txt"),
            sep=",", row.names=F)

write.table(fakedepl,
            file=here::here("the other data","deployments.txt"),
            sep=",", row.names=F)

write.table(fakerange,
            file=here::here("the other data","ranges.txt"),
            sep=",", row.names=F)

write.table(fakedist,
```

```
            file=here::here("the other data","distances.txt"),
            sep=",", row.names=T)
```

Making the detections

```
library(lubridate)

ind1<-sample(seq(as.POSIXct("2020-10-03 23:02:37"), as.POSIXct("2021-03-12 06:58:47"),
                 by="15 mins"),1000)
ind2<-sample(seq(as.POSIXct("2020-10-03 14:02:37"), as.POSIXct("2021-03-12 07:03:13"),
                 by="45 mins"),1000)
ind3<-sample(seq(as.POSIXct("2020-11-01 12:12:31"), as.POSIXct("2021-02-27 07:06:15"),
                 by="30 mins"),1000)
ind4<-sample(seq(as.POSIXct("2020-10-15 14:02:44"), as.POSIXct("2021-04-01 03:03:13"),
                 by="15 mins"),1000)

data<-data.frame(timest=c(ind1[order(ind1)],
                          ind2[order(ind2)],
                          ind3[order(ind3)],
                          ind4[order(ind4)]),
                 recID=c(c(rep(33333, 350),rep(11111,10),rep(33333, 640)),
                         c(rep(33333, 300),rep(11111, 200),rep(22222, 500)),
                         c(rep(11111, 500),rep(22222, 500)),rep(22222, 1000)),
                 transmitID=c(rep(12365, 1000),
                              rep(12366, 1000),
                              rep(12367, 1000),
                              rep(12368, 1000)))

rec1<-data[which(data$recID==11111),]
rec2<-data[which(data$recID==22222),]
rec3<-data[which(data$recID==33333),]

write.table(rec1, file=here::here("the detections","rec1.csv"),
            sep=",", row.names=F)

write.table(rec2, file=here::here("the detections","rec2.csv"),
            sep=",", row.names=F)

write.table(rec3, file=here::here("the detections","rec3.csv"),
            sep=",", row.names=F)
```

Running ATfiltR

```
library(ATfiltR)
```

compileData results in the creation of ATfiltR_data.1

```
compileData(
  detection.folder = "the detections",
  file.ext = ".csv",
  sep.type = ",",
  save = TRUE,
  remove.duplicates = T,
  save.duplicates = F,
  split = T
)
```

```
y
1
y
n
n
n
1
y
y
3
y
y
2
y
y
n
```

```
nrow(ATfiltR_data.1)
```

```
## [1] 4000
```

wWindow results in the creation of ATfiltR_data.2 and out.of.deployment (and unknown.tags which is empty here)

```
wWindow(
  detection.folder = "the detections",
  data.folder = "the other data",
  sep.type = ",",
  save.out.of.deployment = T,
  save.unknown.tags = T,
  discard.first = 1,
  save = T
)
```

```
2
y
3
4
y
1
```

```
y
2
y
y
5
y
2
3
y
1
y
4
y
y
3
y
1
y
2
y
3
y
n
n
y
4
[enter][enter]
y
n
```

```
nrow(ATfiltR_data.2)
```

```
## [1] 3532
```

```
nrow(out.of.deployment)
```

```
## [1] 468
```

As well as all peripheral data reformated for automatic use in *ATfiltR* and saved in the directory under ATfiltR_names

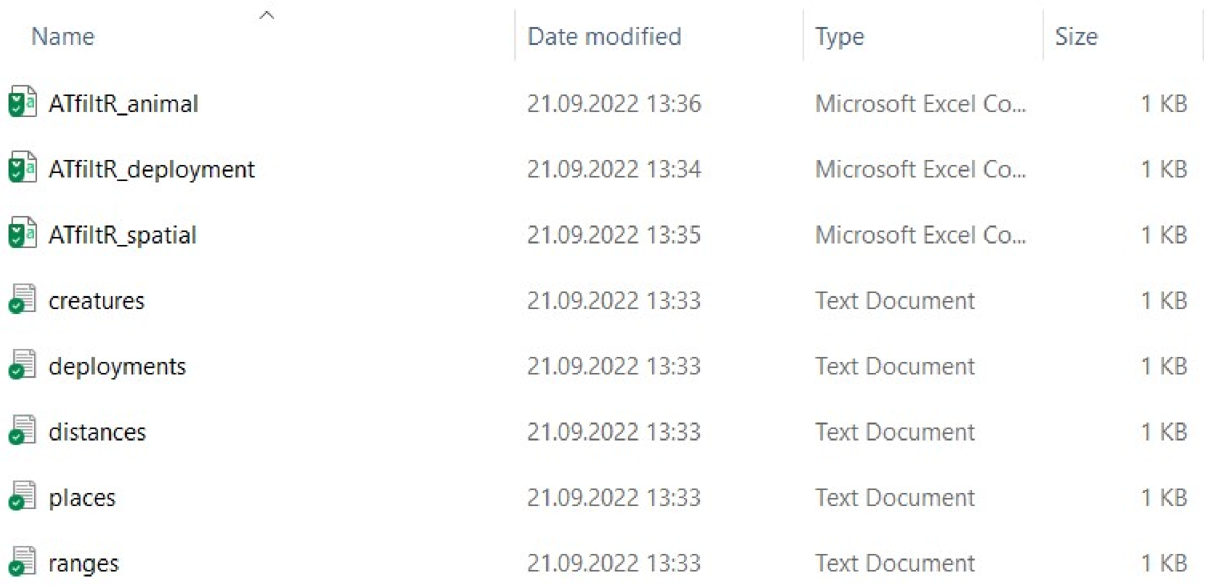

Name	Date modified	Type	Size
ATfiltR_animal	21.09.2022 13:36	Microsoft Excel Co...	1 KB
ATfiltR_deployment	21.09.2022 13:34	Microsoft Excel Co...	1 KB
ATfiltR_spatial	21.09.2022 13:35	Microsoft Excel Co...	1 KB
creatures	21.09.2022 13:33	Text Document	1 KB
deployments	21.09.2022 13:33	Text Document	1 KB
distances	21.09.2022 13:33	Text Document	1 KB
places	21.09.2022 13:33	Text Document	1 KB
ranges	21.09.2022 13:33	Text Document	1 KB

Screenshot of the project directory

findSolo results in the creation of ATfiltR_data.3 and solo.detections

```
findSolo(
  detection.folder = "the detections",
  save.solo = T,
  save = T,
  per.receiver = T,
  delay = 24,
  project = T,
  )
```

```
nrow(ATfiltR_data.3)
```

```
## [1] 3078
```

```
nrow(solo.detections)
```

```
## [1] 454
```

speedCheck results in the creation of ATfiltR_data.4 and speedy.data

```
speedCheck(
  detection.folder = "the detections",
  data.folder = "the other data",
  receiver.range = NA,
  base = 1,
  factor.col = "ship.speed",
  exponent = 1,
  max.distance = NA,
  save.speedy = TRUE,
  save = TRUE
)
```

```
8
y
3
y
y
2
y
"%Y-%m"
y
1
y
6
y
0.01
y
y
```

```
nrow(ATfiltR_data.4)
```

```
## [1] 3052
```

```
nrow(speedy.data)
```

```
## [1] 26
```

Out of the 4000 data points we filtered, 3052 were considered valid!

toActel stores valid actel.detections, biometrics, spatial, and deployments files in the target.folder

```
toActel(
  detection.folder = "the detections",
  data.folder = "the other data",
  target.folder = "for actel"
)
```

```
4
y
```

Name	Date modified	Type	Size
actel.detections	21.09.2022 13:51	R Workspace	84 KB
biometrics	21.09.2022 13:51	Microsoft Excel Co...	1 KB
deployments	21.09.2022 13:51	Microsoft Excel Co...	1 KB
spatial	21.09.2022 13:51	Microsoft Excel Co...	1 KB

Screenshot of the project directory

actel::explore() uses the created files and stores results in the work directory

```
library(actel)
```

```
## Warning: Paket 'actel' wurde unter R Version 4.1.3 erstellt
```

```
## Welcome to actel (1.2.1)!
## Run ?actel for starting tips.
```

```
setwd("~/test ATfiltR/for actel")
```

```
actel::explore(tz = 'Europe/London')
y
y
y
```

Name	Date modified	Type	Size
2022-09-21.13.54.46.actel.log	21.09.2022 13:54	Text Document	3 KB
actel.detections	21.09.2022 13:51	R Workspace	84 KB
actel_explore_results	21.09.2022 13:54	R Workspace	131 KB
biometrics	21.09.2022 13:51	Microsoft Excel Co...	1 KB
deployments	21.09.2022 13:51	Microsoft Excel Co...	1 KB
spatial	21.09.2022 13:51	Microsoft Excel Co...	1 KB

Screenshot of the project directory
